# Retraction notice for: “Schizandrin A exerts anti-tumor effects on A375 cells by down-regulating H19” [Braz J Med Biol Res (2019) 52(10): e8385]

**DOI:** 10.1590/1414-431X2020e8385retraction

**Published:** 2021-07-23

Yiming Bi^1^ https://orcid.org/0000-0002-0436-5301, Yan Fu^2^ https://orcid.org/0000-0003-1740-2498, Shuyan Wang^1^ https://orcid.org/0000-0002-4874-8726, Xingxiu Chen^1^ https://orcid.org/0000-0001-5667-5689, and Xiaoping Cai^1^ https://orcid.org/0000-0001-6433-3435

^1^Department of Oncology, Binzhou People’s Hospital, Binzhou, China

^2^Department of Dermatology, Binzhou People’s Hospital, Binzhou, China

**Retraction for:** Braz J Med Biol Res | doi: 10.1590/1414-431X20198385 | PMID: 31618367 | PMCID: PMC6787960

The authors would like to retract the article “Schizandrin A exerts anti-tumor effects on A375 cells by down-regulating H19” that was published in volume 52, no. 10 (2019) (Epub Oct 10, 2019) of the Brazilian Journal of Medical and Biological Research.

After the publication of this study, the corresponding author requested its retraction due to “the identification of unspecified data inconsistency that could lead to mistaken conclusions.” The Editors agreed with and endorsed that decision.

The Brazilian Journal of Medical and Biological Research had received authorization from all authors before the publication of the paper. We regret the unprofessional behavior of the authors involved.

Correspondence: Yan Fu: <fuyan0059@sina.com>

